# Developing an evidence-based clinical pathway for the assessment, diagnosis and management of acute Charcot neuro-arthropathy

**DOI:** 10.1186/1757-1146-6-S1-P10

**Published:** 2013-05-31

**Authors:** Tamara Milne, Joseph Rogers, Ewan Kinnear, Helen Martin, Peter Lazzarini, Thomas Quinton, Frances Boyle

**Affiliations:** 1 Podiatry Department, Ipswich General Hospital, Ipswich, QLD, 4305, Australia; 2 Podiatry Department, Launceston General Hospital, Launceston, TAS, 7250, Australia; 3 Podiatry Department, The Prince Charles Hospital, Chermside, QLD, 4032, Australia; 4 Allied Health Research Collaborative, The Prince Charles Hospital, Chermside, QLD, 4032, Australia; 5 Prosthetics, Orthotics and Podiatry Department, Princess Alexandra Hospital, Woolloongabba, QLD, 4102, Australia; 6 School of Population Health, University of Queensland, Herston, QLD, 4006, Australia

## Background

Charcot neuro-arthropathy (CN) is one of the most devastating complications of diabetes. To date it appears that no clinical tools based on a systematic review of existing literature have been developed for the management of acute CN. Thus, the aim of this paper was to systematically review existing literature and develop an evidence-based clinical pathway for the assessment, diagnosis and management of acute CN.

## Methods

Electronic databases (Medline, PubMed, CINAHL, Embase, and Cochrane Library), reference lists and applicable websites were systematically searched for literature discussing the assessment, diagnosis and/or management of acute CN. At least two independent investigators then quality rated and graded the evidence of all identified literature. Consistent recommendations emanating from the included literature was then fashioned in a clinical pathway.

## Results

The systematic search identified 267 manuscripts, of which 117 (44%) were assessed to meet the inclusion criteria for this study. As hypothesised, most literature discussing the assessment, diagnosis and/or management of acute CN constituted level IV or EO evidence. The included literature was used to develop an evidence-based clinical pathway for the assessment, investigations, diagnosis and management of acute CN.

## Conclusion

This research has assisted in developing a comprehensive, evidence-based clinical pathway to promote consistent and optimal practice in the assessment, diagnosis and management of acute CN. The pathway aims to support health professionals in making early diagnosis and providing appropriate immediate management of acute CN, ultimately reducing its associated complications such as amputations and hospitalisations.

